# Hospitals with Special Acts of Parliament

**Published:** 1902-03-01

**Authors:** 


### The Hospital

A Journal of

The Medical Sciences and Hospital Administration.

Vol. XXXI.—No. 805.] EDITED BY SIR HENRY BURDETT, K.C.B., AND SOLOMON C. SMITH, M.D., M.R.C.P. [March 1, 1902.

### Hospitals with Special Acts of Parliament.

A question of great interest and importance to all hospitals and institutions incorporated by special Act of Parliament has been solved by the governors of Addenbrooke's Hospital, Cambridge. Addenbrooke's Hospital was incorporated by special Act dated November 11th, 1766. The government of the hospital was by this Act vested in an open court of all the governors meeting weekly, which meetings every governor has a right to attend. Formerly a good many hospitals had this form of government by open board. But as the importance of these institutions increased and their work and influence extended, it became necessary to improve their administrative control by placing the responsibility for their efficiency in the hands of an elected body of governors, *i.e.* a committee or council of administration, limited as to number and renewable by the retirement each year of a fixed proportion of members usually eligible for re-election. The affairs of St. George's Hospital are still managed by an open board, an evidence of the conservatism which dominates its control, for it is the last of the great metropolitan hospitals where this form of administration prevails. There are, besides, a few provincial and county hospitals where the open board is still in force, but everywhere, as in the case of Addenbrooke's Hospital, Cambridge, the desirability of making modifications in the original constitution has become apparent to those who are most familiar with their working and most desirous of adding to the efficiency of their administration.

The governors of Addenbrooke's Hospital, by a substantial majority—145 votes to 95—in November, 1898, decided that a committee of management, consisting of 18 governors, together with a chairman, should be appointed annually, whose duties should include those then assigned to the weekly board, the selected governors and the finance committee. A legal opinion was subsequently obtained to the effect that the appointment of such a committee by the governors was not within the powers conferred by the special Act. In consequence of the expense incidental to a supplemental Act of Parliament, the open Board was not then changed at Addenbrooke's Hospital in accordance with the wishes of the governors expressed at the poll. Since then an advisory council, representing the town, the county and the University of Cambridge, have had the matter under consideration, and a quarterly court, after full discussion, decided on the 19th ult. by a substantial majority to amend the special Act and place the control of the Addenbrooke's Hospital in the hands of a committee of management. This important change, which is calculated to improve the efficiency and add to the income of Addenbrooke's Hospital, whilst largely increasing public interest in the institution, has been rendered possible without the cost of a special Act by the research and industry of the Rev. G. B. Finch, to whom the governors are greatly indebted.

A large number of hospitals and important institutions being incorporated under special Acts of Parliament, the discovery of the Rev. G. B. Finch, whereby important constitutional changes can be made without putting a charity to the expense of a new Act of Parliament, must prove of importance and interest to hospital managers generally. It appears that under the Charitable Trusts Act of 1853 the Charity Commissioners have power, upon the application of the trustees, governors, or other persons concerned in the management of a charity, provided the Commissioners consider it doubtful if such modifications in the constitution can be carried into complete effect otherwise than by the authority of Parliament, to approve and certify a new scheme embodying the necessary modifications of the special Act. It is of course esssential that the Commissioners should satisfy themselves in the first instance that the proposed changes are wise and prudent, and that their adoption is calculated to improve the efficiency of the institution immediately concerned. Subject to this, the Charity Commissioners having approved and sealed such a scheme, it is then reported to the King and laid before both Houses of Parliament, when it may obtain all the authority and force of a public general Act by being scheduled to an Act promoted by the Commissioners or the Government of the day. In this way, by application to the Charity Commissioners under the Charitable Trusts Act of 1853, clauses 54 to 60 inclusive, the governors of any hospital or institution incorporated by special Act of Parliament may amend their special Act without cost or difficulty, should circumstances render such a course desirable at any time.

We anticipate that this old procedure now rediscovered which is so well calculated to benefit Addenbrooke's Hospital, Cambridge, will lead to action on the part of the governors of more than one hospital and institution where modifications in the constitutional government have been desired, in order to bring their practice and administration into line with the best modern methods. There can be no doubt that such necessary reforms, when wisely carried out, tend to awaken fresh interest in old institutions, whereby their usefulness is rejuvenated and the objects of the original founder are best fulfilled. Already important changes resulting from Sir Henry Burdett's report in 1898, have taken place at Addenbrooke's Hospital. We congratulate the governors upon the wise solution, which, after four years' research, they have discovered and decided to apply to their constitutional difficulties. We look forward with confidence to a material improvement in revenue and an increase in reputation for Addenbrooke's Hospital in consequence, which should speedily give it an importance more worthy of the high position which Cambridge occupies amongst the universities of the world, than the humble place hitherto filled by this institution among the hospitals of the Empire.

